# Myocardial PD-L1 Expression in Patients With Ischemic and Non-ischemic Heart Failure

**DOI:** 10.3389/fcvm.2021.759972

**Published:** 2022-01-13

**Authors:** Ekaterina Kushnareva, Vladimir Kushnarev, Anna Artemyeva, Lubov Mitrofanova, Olga Moiseeva

**Affiliations:** ^1^Personalized Medicine Centre, Almazov National Medical Research Centre, Saint Petersburg, Russia; ^2^N. N. Petrov National Medical Research Center of Oncology, Saint Petersburg, Russia; ^3^Non-coronary Heart Disease Department, Almazov National Medical Research Centre, Saint Petersburg, Russia

**Keywords:** ischemic heart disease, dilated cardiomyopathy, myocardial infarction, cardio-oncology, cardiotoxicity, checkpoint, PD-L1

## Abstract

**Objective:** Immune checkpoints inhibitors are promising and wide-spread agents in anti-cancer therapy. However, despite their efficacy, these agents could cause cardiotoxicity, a rare but life-threatening event. In addition, there are still no well-described predictive factors for the development of immune-related adverse events and information on high risk groups. According to known experimental studies we hypothesized that cardiovascular diseases may increase myocardial PD-L1 expression, which could be an extra target for Checkpoint inhibitors and a potential basis for complications development.

**Methods:** We studied patterns of myocardial PD-L1 expression in non-cancer-related cardiovascular diseases, particularly ischemic heart disease (*n* = 12) and dilated cardiomyopathy (*n* = 7), compared to patients without known cardiovascular diseases (*n* = 10) using mouse monoclonal anti-PD-L1 antibody (clone 22C3, 1:50, Dako). Correlation between immunohistochemical data and echocardiographic parameters was assessed. Statistical analyses were performed using R Statistical Software—R studio version 1.3.1093.

**Results:** In the myocardium of cardiac patients, we found membranous, cytoplasmic, and endothelial expression of PD-L1 compared to control group. In samples from patients with a history of myocardial infarction, PD-L1 membrane and endothelial expression was more prominent and frequent, and cytoplasmic and intercalated discs staining was more localized. In contrast, samples from patients with dilated cardiomyopathy displayed very faint endothelial staining, negative membrane staining, and more diffuse PD-L1 expression in the cytoplasm and intercalated discs. In samples from the non-cardiac patients, no convincing PD-L1 expression was observed. Moreover, we discovered a significant negative correlation between PD-L1 expression level and left ventricular ejection fraction and a positive correlation between PD-L1 expression level and left ventricular end-diastolic volume.

**Conclusions:** The present findings lay the groundwork for future experimental and clinical studies of the role of the PD-1/PD-L1 pathway in cardiovascular diseases. Further studies are required to find patients at potentially high risk of cardiovascular adverse events associated with immune checkpoint inhibitors therapy.

## Introduction

Programmed cell death receptor 1 (PD-1) and its ligand PD-L1 are involved in the regulation of T-cell activation, tolerance, and immune-mediated organ damage. Under physiological conditions, PD-1/PD-L1 signaling plays an important role in the prevention of autoimmune diseases. Apart from the expression on T- and B-cells, dendritic cells, and macrophages, PD-L1 could be expressed on non-hematopoietic cells, including cardiomyocytes and endothelial cells. Recently, it was found that a wide range of tumor cells express PD-L1 on their surface to prevent antitumor immune response (1). As a result, a new strategy for the treatment of advanced or metastatic cancer based on inhibition of PD-1 on the surface of T-cells or blocking PD-L1 on the tumor cells surface has appeared.

Therapy with immune checkpoint inhibitors (ICI) was associated with increased overall survival in patients with advanced cancer that previously had a poor prognosis. The results of KEYNOTE-042 trial comparing the effectiveness of ICI and standard chemotherapy in patients with advanced or metastatic non-small cell lung cancer (NSCLC) and PD-L1 tumor proliferation score (TPS) >50% showed that relapse-free survival was better in a group of ICI therapy−20 months vs. 12.2 months in the standard chemotherapy group (2). The KEYNOTE-522 trial confirmed the higher efficiency of combination therapy with pembrolizumab–chemotherapy against the placebo–chemotherapy group in patients with triple-negative breast cancer, as measured by relapse-free survival and a pathological complete response at the time of definitive surgery (3). The long-term outcomes of ICI therapy were measured for the CheckMate-017 and 057 trials and assessed 5-years overall survival and safety. Overall survival was longer in NSCLC patients receiving nivolumab than in NSCLC patients on chemotherapy (13.4% vs. 2.6%), and treatment-related adverse events were found in 25.8% of nivolumab-treated patients (4).

Cardiotoxic side-effects of ICI therapy have been reported since 2016. First publications described the development of fulminant myocarditis in patients receiving ICI (5–7). Moreover, there were cases of myopericarditis, takotsubo-like syndrome, and vasculitis with acute coronary syndrome symptoms (8–11). However, the true incidence of immune-related adverse events (irAEs) is still unknown and, according to some data, is in the range of 1 to 10.3% (12, 13). On the other hand, ICI-related myocarditis, one of the most common cardiac irAEs, has a relatively high mortality rate of 40–50% (14, 15).

Currently, there are no methods to identify patients at high risk for the development of ICI-associated cardiotoxicity. Moreover, the impact of pre-existing cardiovascular (CV) disease and traditional CV risk factors in cardiac irAEs occurrence is not yet fully understood. Histological and immunohistochemical analysis revealed high levels of membrane and cytoplasmic PD-L1 expression in samples from patients with ICI-associated myocarditis (5, 16). However, the role of PD-1/PD-L1 signaling in the development of non-cancer-related CV diseases is unclear. *In vivo* experiments performed by Grabie et al. discovered that IFN-γ-induced PD-L1 was mainly expressed on endothelium and its expression had an important cardioprotective effect against immune-related heart damage (17). Later, it was shown that PD-L1–/– knockout mice had a higher risk for the development of autoimmune myocarditis and pneumonitis with a more severe course of the disease and worse prognosis compared to PD-L1+/– and PD-L1+/+ animals (18). Baban et al. showed that in the model of ischemia-reperfusion injury and cryoinjured hearts, PD-L1 expression was markedly higher than in intact cells (19).

Up-regulation of PD-L1 may probably attenuate T-cell response against damaged cardiomyocytes, for example, in the course of ischemic heart disease (IHD), thus reducing the local inflammation in the myocardium. On the other hand, high PD-L1 myocardial expression in CV diseases might be associated with an increased risk of developing irAEs, since PD-L1 is a direct target for anti-PD-1 and anti-PD-L1 ICI. However, there is still no research demonstrating increased myocardial expression of PD-L1 in damaged human hearts due to different types of CV diseases.

To characterize the PD-L1 expression pattern in patients with CV diseases of different etiology, we analyzed PD-L1 myocardial expression in patients with documented IHD and dilated cardiomyopathy (DCM).

## Materials and Methods

**Figure 1:**
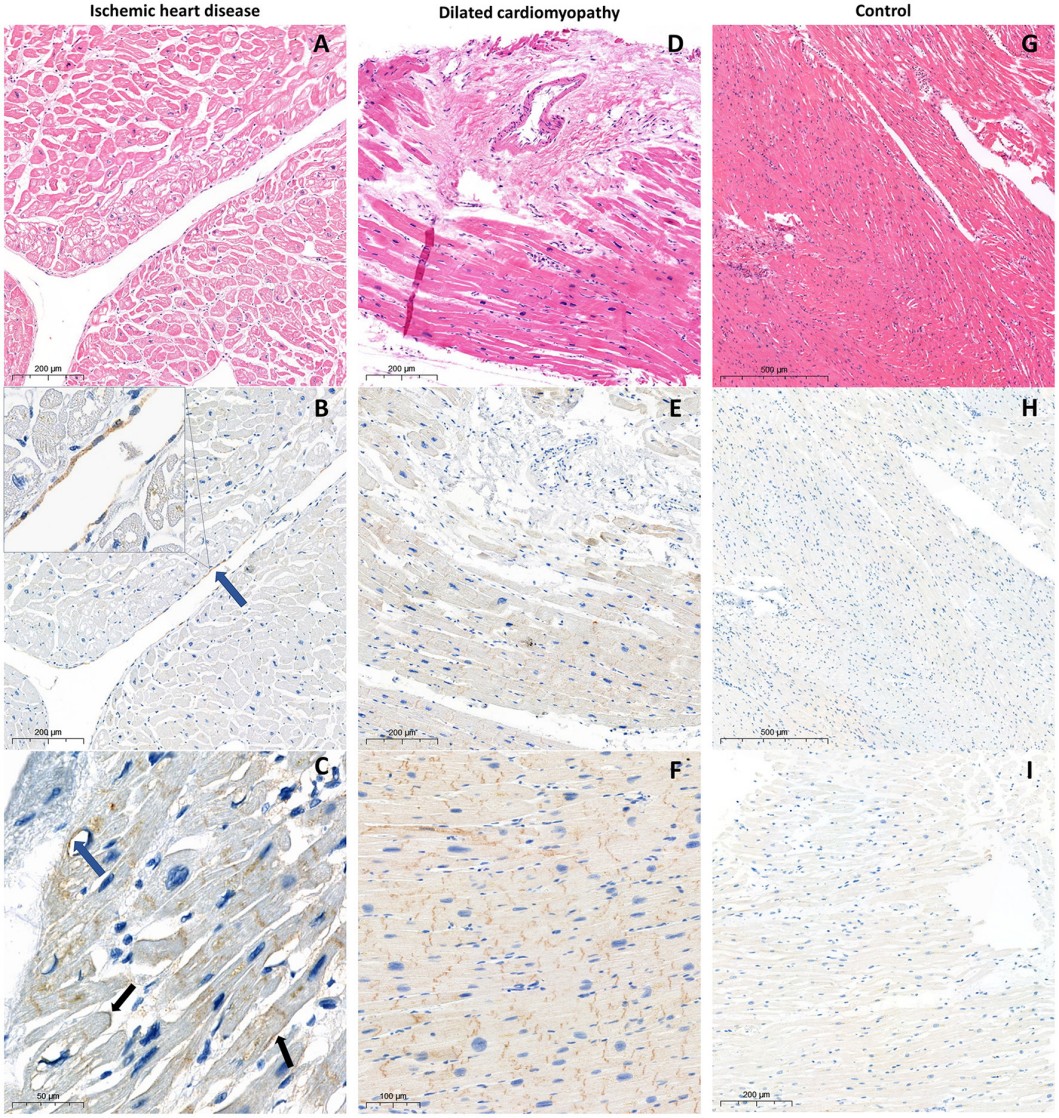
Histological and immunohistological examination of the myocardium samples from the patient with ischemic heart disease **(A)** hematoxylin and eosin staining; **(B,C)** PD-L1 staining with CMPS 5% and 20% respectively. Blue arrows indicate positive endothelial PD-L1 expression, black arrows indicate positive membrane PD-L1 expression; from the patient with dilated cardiomyopathy **(D)** hematoxylin and eosin staining; **(E,F)** PD-L1 expression in cytoplasm and intercalated discs, without endothelial, perivascular, and membrane patterns with ICDPS 70% and 100% respectively; from the control without ICI treatment **(G)** hematoxylin and eosin staining; **(H)** absence of PD-L1 expression; **(I)** extremely poor cytoplasmic PD-L1 expression.

We examined 12 autopsy samples of left ventricular (LV) myocardium obtained from patients with a history of myocardial infarction (MI). Nine patients died in an acute period of MI. Cardiac pathology specimens from an infarct-related artery were used for further immunohistochemical evaluation. The comparison group included seven samples of LV from patients with DCM who underwent orthotopic heart transplantation. In this group, IHD was excluded according to coronary angiography results. Echocardiography was carried out for all patients at one clinic. The control group included 10 LV samples collected from cancer patients without known CV pathology who died in the early postoperative period and had not received neoadjuvant chemotherapy and/or immunotherapy and 4 LV samples from patients received ICI and died without intravital data for CV irAEs (two without CV diseases and two with known IHD). The tissue was fixed in 10% neutral buffered formaldehyde and then embedded in paraffin. We used hematoxylin and eosin (HE) staining to visualize the myocardial structure and immunohistochemistry to investigate the expression of PD-L1 and distribution of CD3^+^ T-cells and CD68+ macrophages. Additionally, the correlation analysis between echocardiographic parameters, complete blood count and histological results has been conducted (Figures 1A,D,G).

### Ethics Approval

The study was approved by the local ethics committee (Protocol Number: 12032020 of March 16, 2020).

### PD-L1 Expression and T-Cells Immune Infiltration Assessment

Immunohistochemistry was performed on the automated immunostaining platform Autostainer Link 48 (Dako, USA) for PD-L1 and Ventana Benchmark Ultra (Roche, Switzerland) for CD3. Tissue sections were immunostained with mouse monoclonal anti-PD-L1 antibody (clone 22C3, 1:50, Dako), rabbit monoclonal anti-CD3 antibody (clone 2GV6, Ventana) and mouse monoclonal anti-CD68 antibody (KP1, Abcam). All slides were scanned using a Pannoramic 1000 scanning microscope (3D Histech) with a x60 objective lens. Assessment of PD-L1 was performed by an experienced board pathologist. CD3 and CD68 expression was quantify with digital image analysis of scanned by QuPath software. We assessed membrane, cytoplasmic, and endothelial PD-L1 expression in all groups.

To characterize the expression level of PD-L1 in the myocardium, we developed a combined cardiomyocyte positive score (CMPS). CMPS was calculated as a percentage of PD-L1 positively stained cardiomyocytes with membrane and/or cytoplasmic expression of any intensity. To additionally evaluate the PD-L1 expression in intercalated discs (ICD), we determined the PD-L1 ICD positive score (ICDPS), which was defined as a percentage of positively stained ICD from all cardiomyocytes cut in a longitudinal section.

### Statistical Analysis

Data were expressed as mean and standard deviation (Mean ± SD) or median with 25th and 75th percentiles (Median [25;75]). Clinical and expression data were analyzed using the Mann-Whitney U test for continuous variables and Fisher Exact test for dichotomous variables. Correlations were calculated with Spearman's rank correlation coefficient for non-parametric samples. *p*-values <0.05 were considered significant. All statistical analyses were performed using R Statistical Software—R studio version 1.3.1093.

## Results

The mean age in the MI group at the time of death was 66.1 ± 7.0 years. For patients who died from acute MI (*n* = 9), the mean time interval between symptom onset and death was 7.9 ± 4.3 days. During hospitalization, 10 of 12 patients underwent percutaneous transluminal coronary angioplasty, and one patient underwent coronary artery bypass graft surgery. The mean age in the DCM group at the time of heart transplantation was 52.1 ± 9.8 years, which was significantly lower than in the MI group (*p* = 0.008**)**. There were no age differences between the experimental and control groups. Control group age was 59.5 ± 12.4 (*p* = 0.197 for MI and *p* = 0.186 for DCM). Clinical, echocardiography, and immunohistochemistry data of studied groups (MI and DCM) are summarized in Table 1.

**Table 1:** Clinical, echocardiographic, and immunohistochemical characteristics of patients.

	**IHD (*n* = 12)**	**DCM (*n* = 7)**	***p***
Age, years	66.1 ± 7.0	52.1 ± 9.8	**0.008**
Male sex, n (%)	12 (100)	4 (57.1)	0.361
LVEF, %	34.9 ± 7.2	20.3 ± 7.1	**0.005**
LVEDV, ml	194.3 ± 64.2	275.0 ± 82.2	0.071
Membrane PD-L1, n (%)	5 (41.7)	0 (0)	0.068
Cytoplasmic PD-L1, n (%)	10 (83.3)	7 (100)	0.386
Endothelial PD-L1, n (%)	4 (33.3)	0 (0)	0.127
ICD PD-L1, n (%)	7 (58.3)	7 (100)	0.068
PD-L1 ICDPS, %	1 [0;32.5]	90 [85;100]	**0.003**
PD-L1 CMPS, %	10 [5;17.5]	90 [30;100]	**0.001**

*IHD, ischemic heart disease; DCM, dilated cardiomyopathy; LVEF, left ventricular ejection fraction; LVEDV, left ventricular end diastolic volume; ICD, intercalated discs; CMPS, cardiomyocyte positive score. Bold values are p < 0.05*.

In all patients from the DCM group, according to histopathological evaluation, <7 CD3^+^ T-cells per mm^2^ were detected [4 (3; 5) cells per mm^2^], so inflammation cardiomyopathy was excluded (20). The median number of CD3^+^ T-cells in MI group was 15.3 [8; 19] cells per mm^2^ and 62 [50,5;93] cells per mm^2^ for CD68.

**Figure 2:**
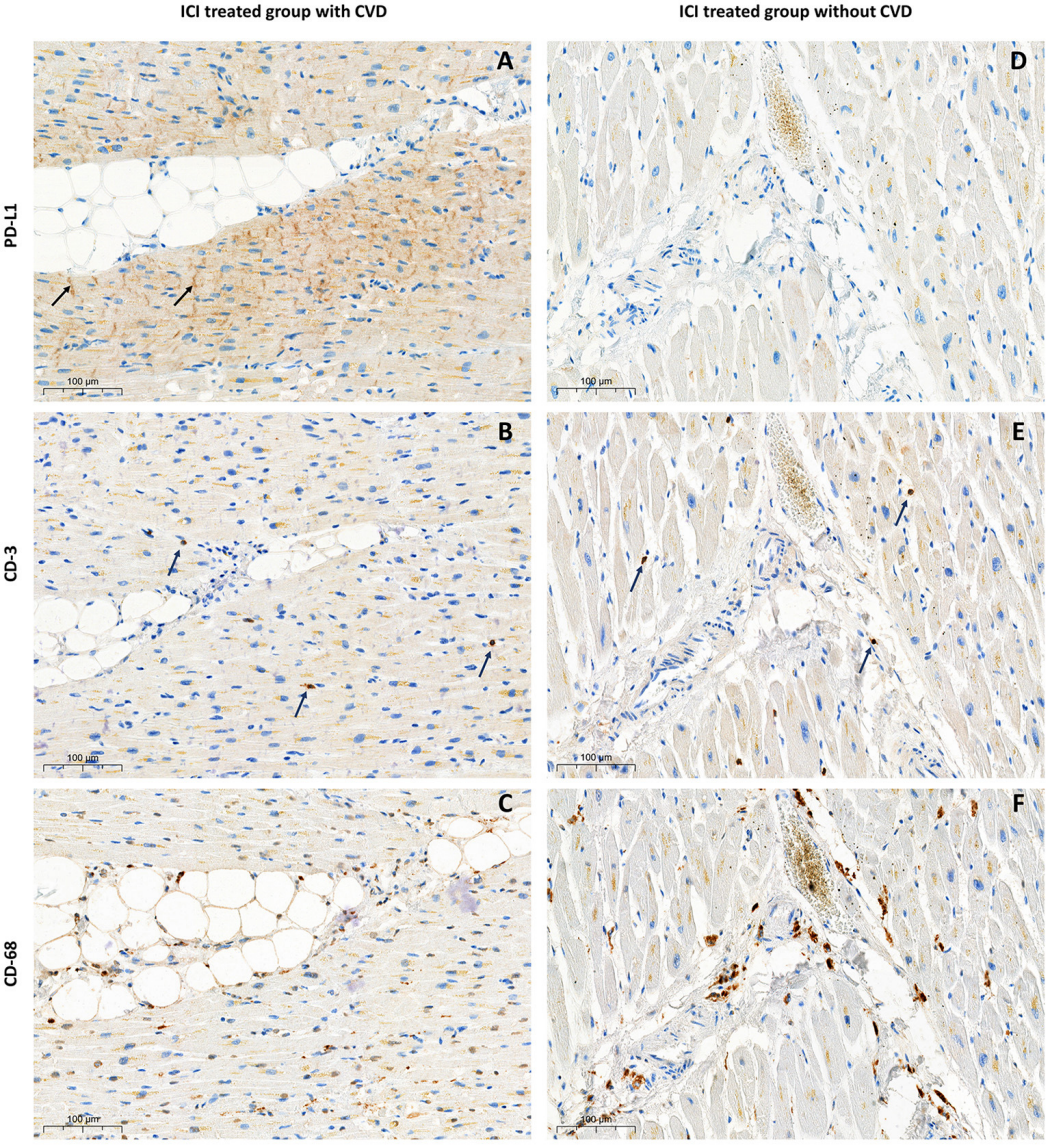
Immunohistological examination of the myocardium samples from patients treated with ICI. With pre-existing CVD—left column **(A)** PD-L1 expression in ICD with CMPS 50%, ICDPS 40%; **(B)** CD-3 infiltration 7 cells per mm^2^; **(C)** CD-68 infiltration 142 cells per mm^2^; Without pre-existing CVD—right column **(D)** absence of PD-L1 expression; **(E)** CD-3 infiltration 17 cells per mm^2^; **(F)** CD-68 infiltration 92 cells per mm^2^.

In both studied groups, according to immunohistochemical evaluation, cytoplasmic and ICD PD-L1 expression was found. Membrane and endothelial PD-L1 expression was identified only in patients with ischemic myocardial damage. Furthermore, in this group, cytoplasmic and membrane PD-L1 expression in perivascular zones was more pronounced (Figures 1B,C,E,F). In samples from the control group without ICI treatment, there was a lack of membrane, endothelial, and ICD expression, accompanied by infrequent cytoplasmic PD-L1 expression, which appears to be a non-specific finding (Figures 1H,I). In the control group receiving ICI pronounced PD-L1 expression was found only in samples with pre-existing CVDs (*n* = 2; CMPS = 50 and 70%; ICDPS = 40 and 20%). In contrast, PD-L1 expression was not detected in patients without CVDs in ICI therapy group (Figure 2). The median number of CD3 and CD68 cells in control ICI group was 17 [11;102] and 149 [129;180] cells per mm^2^ respectively.

There were no statistically significant differences between experimental groups in the presence or absence of different expression patterns. However, DCM group had significantly higher CMPS (90 [30;100] vs. 10 [5;17.5], *p* = 0.001) and ICDPS (90 [85;100] vs. 1 [0;32.5], *p* = 0.003) compared to the MI group.

**Figure 3:**
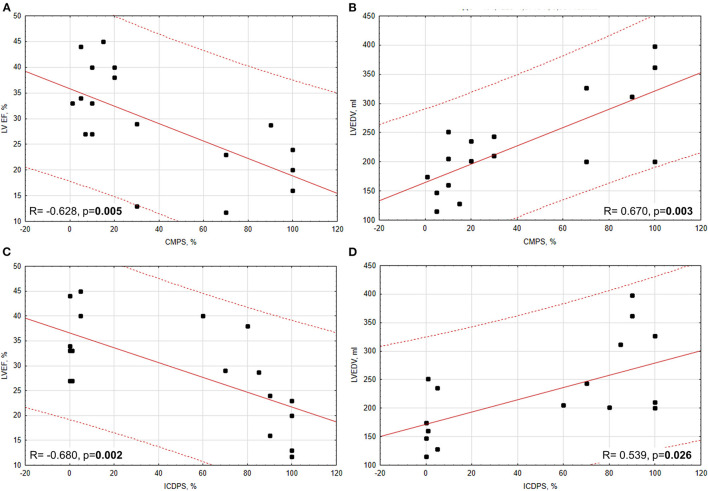
Correlations between PD-L1 expression (CMPS, ICDPS) and echocardiographic parameters (LVEF and LVEDV). **(A)** Significant moderate negative correlation between CMPS and LVEF; **(B)** significant moderate positive correlation between CMPS and LVEDV; **(C)** significant moderate negative correlation between ICDPS and LVEF **(D)** significant moderate positive correlation between ICDPS and LVEDV.

According to correlation analysis between immunohistochemistry and echocardiography data, we got the following results (Figure 3). In all patients with CV diseases (IHD + DCM), there were significant negative correlations between CMPS and LVEF (*R* = −0.628, *p* = 0.005) and between ICDPS and LVEF (*R* = −0.680, *p* = 0.002), and significant positive correlations between CMPS and LVEDV (*R* = 0.670, *p* = 0.003) and between ICDPS and LVEDV (*R* = 0.539, *p* = 0.026). After dividing patients into subgroups, a significant negative correlation between ICDPS and LVEF (*R* = −0.861, *p* = 0.013) remained only in the DCM group. In the group of MI, only the tendency to a positive correlation between ICDPS and LVEDV was found (*R* = 0.605, *p* = 0.064). The lack of other significant correlations between studied parameters in subgroups may be attributed to a low number of analyzed samples.

Also, we have indicated strong positive correlations between myocardial PD-L1 expression (CMPS, ICDPS) and complete blood count (WBC, neutrophils) for patients with IHD (Supplementary Figure 1). No correlation between PD-L1 expression and monocytes or lymphocytes count was found. In DCM group PD-L1 expression didn't correlate with complete blood count data.

## Discussion

The present study aimed to examine the expression profiles of PD-L1 in the myocardium of cardiac patients without a history of cancer. In damaged myocardium from patients with IHD and DCM, we found several patterns of PD-L1 expression compared to the myocardium of patients without CV diseases. The presence of membrane and endothelial expression was more specific for patients with MI history than those without ischemic damage. The reason for this result is not yet fully understood. Previously, PD-L1 endothelial expression was described in the mouse model of CD8 T-cell myocarditis (17). Therefore, endothelial expression we found in ischemic injured myocardium could be caused by chronic inflammation, which is evidenced by an increase in the number of CD3+ cells.

Further analysis revealed PD-L1 expression in intercalated discs in all groups of cardiac patients, but predominantly PD-L1 was observed in the DCM group. This matches well with the recently described strong but diffuse staining of PD-L1 in ICD of cardiac allograft vasculopathy hearts. However, the staining was considered by the authors as non-specific and insignificant (21).

The crucial role in ICI toxicity development plays activation of inflammatory response which damage tissues and organs. Cytokines that are secreted by immune cells such as macrophages, activated T-cells, B-cells and NK cells take a lead in irAEs pathophysiology. Increased levels of IFN-γ and IFN-γ pathway genes are positive biomarkers of tumor response on ICI treatment and irAEs and IL-8, IL-6, and TGF-β are negative biomarkers (22). Experimental study evaluated cardiomyocyte cell line showed an increase of IL-1β, IL-8, IL-6, and TGF-β after Nivolumab and Ipilimumab affection (23). There are no experimental studies described features of cytokines levels and PD-L1 expression in human hearts after ICI administration. We showed PD-L1 expression in ICD in patients with history of CVD treated by ICI with significantly more pronounced CD-68 infiltration compared with those who had CVD but didn't receive ICI (*p* = 0.01). The limitation of our study is that we used archived material presented by paraffin blocks which makes it impossible to conduct flow cytometry or ELISA assay to describe T-cell immunophenotyping and cytokines levels.

In large retrospective study Oren et al. showed the increasing of ICI-related myocarditis risk from 0.13% to 4.5% in patients with history of MI, HF and age >80 years (24). But the mechanism of such risk increasing is unknown. In our studied groups, we found a negative correlation of PD-L1 expression prevalence in ICD, calculated as ICDPS, and LVEF and a positive correlation between ICDPS and LVEDV. These results may partially explain previously published clinical data. LV dilation occurred due to ischemia or cardiomyopathy likely resulted in disruption of intercellular contacts. Thus, PD-L1 expression can be considered as one of the possible cardioprotective mechanisms against myocardial injury. Also, there is experimental study shown that hyperglycemia increased cardiomyocyte damage during anti-CTLA4 ICI (Ipilimumab) administration (25). Another clinical study showed that diabetes is associated with an increase in PD-L1 positivity and recurrence in NSCLC (26). But there is no experimental data about direct anti-PD-L1 treatment influence on cardiomyocyte damage in condition of hyperglycemia. Nevertheless, based on the known data we may hypothesize that known CV comorbidity with diabetes may be a combined risk factor in patient treated with anti-CTLA4+anti-PD-1 immunotherapy. But to prove it another experimental and clinical investigations are needed.

To sum up, our work described an increase of PD-L1 expression in the myocardium of cardiac patients and revealed a correlation between PD-L1 expression (CMPS, ICDPS) and echocardiographic parameters of left ventricular size and function (LVEDV and LVEF). The findings of this study lay the groundwork for further investigations aimed to identify the high risk patients for CV irAEs and give us a reason to pay more attention to patients with LV dysfunction and heart chambers enlargement.

We are aware that our research may have the limitation of a small sample size that did not allow us to investigate additional correlations in distinct subgroups of cardiac patients.

## Data Availability Statement

The raw data supporting the conclusions of this article will be made available by the authors, without undue reservation.

## Ethics Statement

The study was approved by the local ethics committee (Protocol Number: 12032020 of March 16, 2020). The patients/participants provided their written informed consent to participate in this study.

## Author Contributions

EK performed data collection, statistical analyses, and wrote manuscript. VK performed immunohistochemical analysis. AA, LM, and OM provided the critical revision of the manuscript. All authors contributed to the article and approved the submitted version.

## Funding

This study has been supported by the grant from the Ministry of Science and Higher Education of the Russian Federation (Agreement No. 075-15-2020-901).

## Conflict of Interest

The authors declare that the research was conducted in the absence of any commercial or financial relationships that could be construed as a potential conflict of interest.

## Publisher's Note

All claims expressed in this article are solely those of the authors and do not necessarily represent those of their affiliated organizations, or those of the publisher, the editors and the reviewers. Any product that may be evaluated in this article, or claim that may be made by its manufacturer, is not guaranteed or endorsed by the publisher.

## Supplementary Material

Supplementary Figure 1 Correlations between PD-L1 expression (CMPS, ICDPS) and white blood cells (WBC) count in peripheral blood. **(A)** Significant strong positive correlation between CMPS and WBC; **(B)** significant strong positive correlation between CMPS and neutrophils; **(C)** significant strong positive correlation between ICDPS and WBC; **(D)** significant strong positive correlation between ICDPS and neutrophils.
