# Supplementary figures and images for: Myocardial PD-L1 Expression in Patients With Ischemic and Non-ischemic Heart Failure
Source: Front Cardiovasc Med. 2022 Jan 13;8:759972. doi: 10.3389/fcvm.2021.759972 (PMC8792535; doi:10.3389/fcvm.2021.759972)

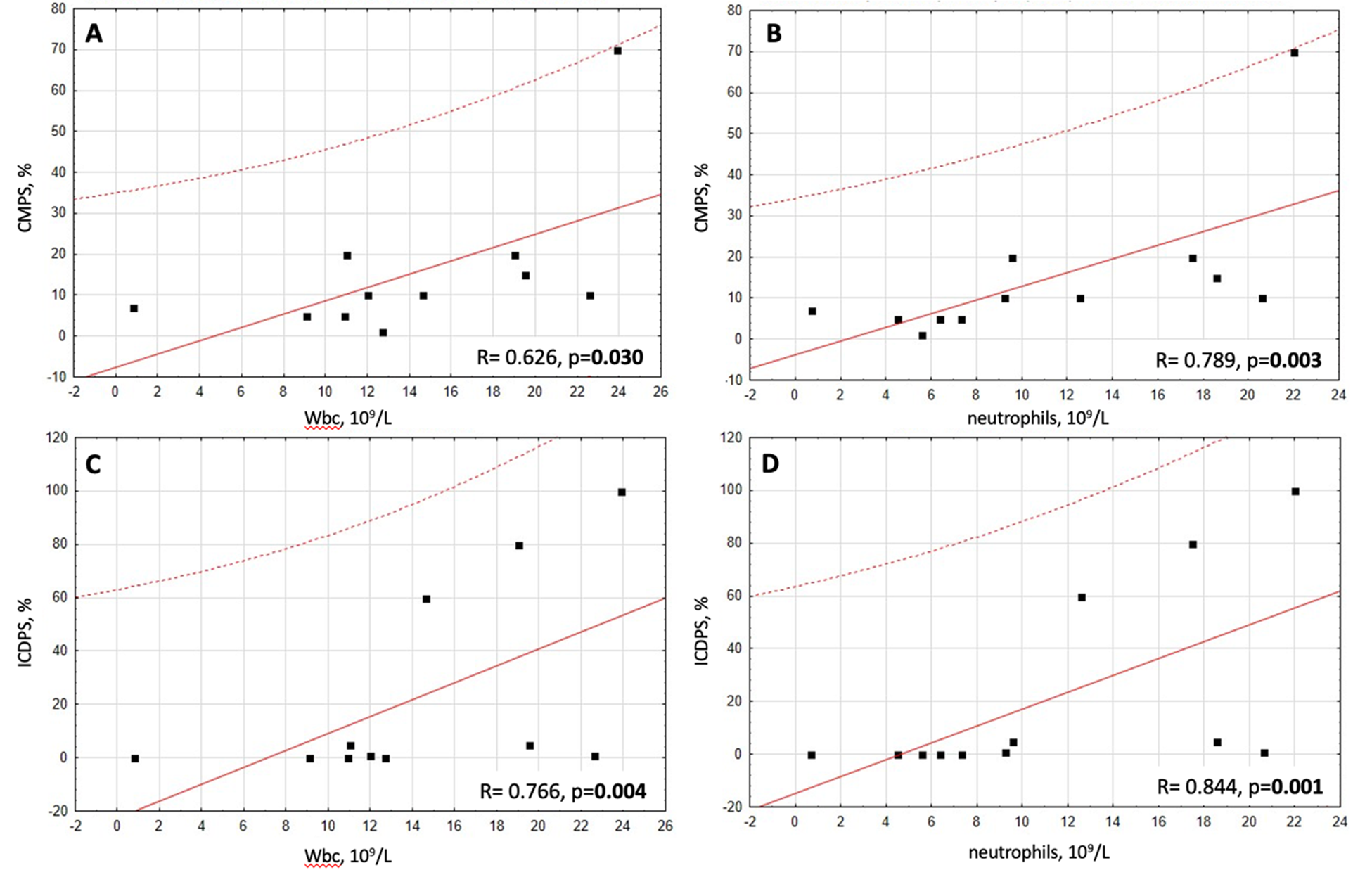

Supplement: Supplementary Figure 1 — Correlations between PD-L1 expression (CMPS, ICDPS) and white blood cells (WBC) count in peripheral blood. (A) Significant strong positive correlation between CMPS and WBC; (B) significant strong positive correlation between CMPS and neutrophils; (C) significant strong positive correlation between ICDPS and WBC; (D) significant strong positive correlation between ICDPS and neutrophils. [file Image_1.TIF]
